# Emapunil attenuates ulcerative colitis by suppressing Z-DNA binding protein 1 driven pyroptosis and pro-inflammatory polarization in macrophages

**DOI:** 10.3389/fimmu.2026.1813664

**Published:** 2026-05-26

**Authors:** Shenghao Xv, Jie Hao, Sanhua Deng, Zhengyin Zhang, Runshu Wang, Jianbin Yin, Peisheng Chen, Fengjian He, Qianqian Peng, Fang Xie, Erwei Sun, Shimin Zheng

**Affiliations:** 1 Department of Gastroenterology and Hepatology, The Third Affiliated Hospital, Southern Medical University, Guangzhou, China; 2 Guangdong Provincial Key Laboratory of Bone and Joint Degeneration Diseases, The Third Affiliated Hospital, Southern Medical University, Guangzhou, Guangdong, China; 3 Department of Rheumatology and Immunology, The Third Affiliated Hospital, Southern Medical University, Guangzhou, China; 4 The Third School of Clinical Medicine, Southern Medical University, Guangzhou, China

**Keywords:** emapunil, macrophage polarization, pyroptosis, ulcerative colitis, Z-DNA binding protein 1

## Abstract

**Background:**

The pathogenesis of ulcerative colitis (UC) remains incompletely understood, and effective therapeutic targets are still lacking in clinical practice. Macrophage pyroptosis and polarization imbalance are core events in UC progression. As a key upstream regulator of pyroptosis, the role of Z-DNA binding protein 1 (ZBP1) in UC-associated macrophages has not been systematically elucidated. Our previous study demonstrated that Emapunil could suppress macrophage inflammation and downregulate ZBP1 expression, suggesting its potential as a candidate agent for UC treatment.

**Methods:**

Immunofluorescence was used to detect the expression and localization of ZBP1 in colorectal tissues from UC patients and DSS-induced colitis mice. ZBP1 overexpression, knockdown, and Emapunil intervention were performed in macrophages to analyze their effects on pyroptosis, polarization, NF-κB pathway, and intestinal barrier function. The therapeutic effect and mechanism of Emapunil on UC were verified via *in vitro* and *in vivo* experiments.

**Results:**

ZBP1 was significantly upregulated in colorectal macrophages from UC patients. Overexpression of ZBP1 induced macrophage pyroptosis and M1 polarization, activated the NF-κB pathway and impaired intestinal barrier integrity, whereas ZBP1 knockdown markedly reversed these effects. By targeting TSPO, Emapunil effectively suppressed macrophage pyroptosis and inflammatory polarization via downregulating ZBP1, alleviated DSS−induced colonic injury, and preserved intestinal barrier function in mice.

**Conclusion:**

ZBP1 aggravates intestinal inflammation and barrier damage by driving macrophage pyroptosis and pro-inflammatory polarization, representing a novel key molecule regulating abnormal macrophage activation in UC. By targeting TSPO, Emapunil exerts anti−UC effects through downregulation of ZBP1, thereby providing a novel mechanism and a potential therapeutic strategy for the clinical management of ulcerative colitis.

## Introduction

Ulcerative colitis (UC), a major subtype of inflammatory bowel disease (IBD), is a chronic idiopathic inflammatory disorder of the gastrointestinal mucosa (1). Despite recent advances in targeted therapies such as biologics, its pathogenesis remains incompletely elucidated and effective therapeutic strategies remain limited. Clinical management continues to face challenges, including limited remission rates and a high propensity for relapse.

In recent years, the role of programmed cell death in inflammatory diseases has attracted considerable attention. Pyroptosis is a pro-inflammatory form of programmed cell death that is mediated by gasdermin family proteins and activated by inflammasomes; it plays crucial roles in host defence and inflammatory regulation (2, 3). Pyroptosis is accompanied by the release of large amounts of pro-inflammatory cytokines such as interleukin (IL)-1β and IL-18 and the formation of plasma membrane pores, which can exacerbate local inflammatory responses and tissue damage (4, 5). A growing body of evidence indicates that excessive inflammasome activation and upregulated pyroptosis−related protein expression have been observed in both the colorectal mucosa of patients with UC and in experimental models of colitis (6–8). This suggests that aberrant pyroptosis of intestinal epithelial cells and immune cells may contribute to intestinal barrier disruption, dysregulated immune activation and a persistent inflammatory state in UC.

Z-DNA-binding protein 1 (ZBP1), also known as DAI or DLM-1, is a cytosolic protein that serves as an upstream core innate immune sensor and key initiator of pyroptosis (9). ZBP1 specifically recognises aberrant endogenous or pathogen-derived Z-form nucleic acids and activates the NOD-like receptor thermal protein domain associated protein 3 (NLRP3) inflammasome to induce Caspase1 cleavage into its p20 active form; this active form in turn cleaves gasdermin D (GSDMD) to generate its N-terminal fragment (GSDMD-NT) that mediates pyroptotic plasma membrane pore formation and ultimately triggers pyroptotic cell death. Meanwhile, this NLRP3 inflammasome activation promotes the maturation and release of pro-inflammatory cytokines such as IL-1β (10, 11). These findings highlight the important role of ZBP1 in inflammatory responses.

ZBP1 is widely recognized as a critical innate immune regulator that modulates the function of multiple immune cell types in a cell−specific manner. In B cells, ZBP1 is selectively expressed during late developmental stages and is essential for T cell−dependent humoral immune responses (12). In natural killer cells, ZBP1 deficiency enhances cytotoxic activity and indicates an inhibitory regulatory role (13). ZBP1 also exhibits non−classical functions in plasma cells and participates in CD8^+^ T cell−mediated antitumor immunity (14). Given its broad immune regulatory functions, the role of ZBP1 in macrophages, which are central immune effectors in intestinal inflammation, remains poorly understood.

Studies have reported the elevated expression of ZBP1 in colorectal epithelial cells from patients with UC (15, 16). However, research on ZBP1 in UC remains limited and has primarily focused on epithelial cells. Its role in immune cells, particularly macrophages, remains unclear. Macrophages play central roles in initiating, sustaining and resolving inflammation in IBD (17, 18). Activated macrophages can polarise into pro-inflammatory M1 macrophages, which release large amounts of cytokines such as tumour necrosis factor (TNF)-α, IL-1β and IL-6 to directly induce tissue damage and impair the integrity of the intestinal epithelial barrier (19, 20). Although recent studies have shown that macrophage pyroptosis occurs in UC and promotes pro-inflammatory responses by driving M1 polarization (21, 22), the role and mechanism of the ZBP1-mediated pyroptosis pathway in UC macrophages remain to be systematically investigated.

Our previous transcriptomic sequencing analysis revealed that Emapunil treatment could inhibit the inflammatory responses of lipopolysaccharide (LPS)-stimulated macrophages and significantly downregulate ZBP1 expression. Pathway enrichment analysis indicated a close association with signaling pathways related to IBD. Emapunil (XBD-173) is an orally available, selective ligand of 18-kDa mitochondrial translocator protein (TSPO). Originally developed for its anxiolytic potential, Emapunil has demonstrated notable anti-inflammatory and neuroprotective effects in preclinical models of neurological disorders (23, 24) and has been shown to suppress inflammation in osteoarthritis by modulating macrophage function (25). We therefore hypothesise that Emapunil may alleviate UC by regulating ZBP1 in macrophages.

In this study, we confirmed that ZBP1 expression is upregulated in UC colorectal tissues and that this upregulation is more pronounced in macrophages than in other cell types. High expression of ZBP1 induced pyroptosis in macrophages and promoted their differentiation towards a pro-inflammatory M1 phenotype, thereby disrupting the colorectal epithelial barrier function. Emapunil effectively downregulated ZBP1 expression in macrophages, inhibited macrophage pyroptosis and M1 polarization and exerted protective effects on the colorectal epithelial barrier in both *in vivo* and *in vitro* models. Therefore, ZBP1 and Emapunil show promise as a potential novel therapeutic target and a candidate drug for UC treatment, respectively.

## Materials and methods

### Clinical human samples

This study was approved by the Ethics Committee of the Third Affiliated Hospital of Southern Medical University. Newly diagnosed patients with active UC aged 18–65 years and age-matched healthy controls were enrolled. UC patients had a total Mayo Score (26) ≥ 6 and an Mayo Endoscopic Subscore ≥ 1, while healthy controls showed no inflammation or structural abnormalities confirmed by colonoscopy and pathological examination. Subjects with prior UC-related treatment, other autoimmune or infectious diseases, and long-term use of non-steroidal anti-inflammatory drugs, aspirin, or immunosuppressive agents were excluded. After obtaining informed consent from all participants meeting the inclusion and exclusion criteria, colorectal tissues were collected from 12 UC patients (age 36.3 ± 7.4 years; 7 males, 5 females) and 12 individuals with no history of colorectal diseases undergoing colonoscopy for health screening (age 36.3 ± 5.6 years; 6 males, 6 females). The clinical characteristics of the patients are presented in **Supplementary Table 1**. All samples were uniformly collected by designated personnel.

### Animal experiment

**Figure 1:**
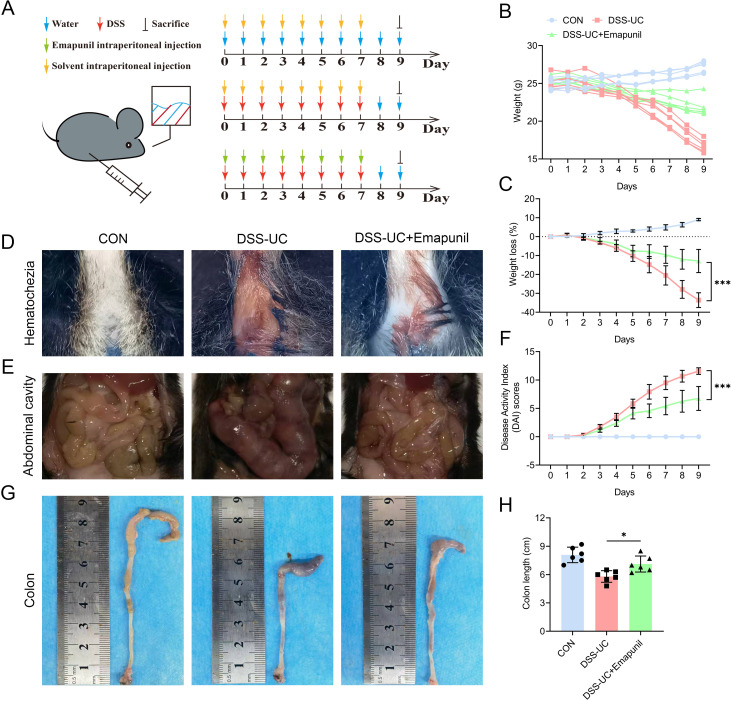
Emapunil ameliorates DSS-induced colitis in mice. **(A)** Schematic diagram of the experimental design for animal model. **(B)** Body weight changes of mice in the CON, DSS-UC and DSS-UC+Emapunil groups over the 9-day experimental period (n = 6 per group). *P < 0.05, **P < 0.01, ***P < 0.001, ns: not significant. Data are presented as mean ± SD. Statistical analyses were conducted using two-way ANOVA followed by Tukey’s *post-hoc* test. **(C)** Percentage of body weight loss in the CON, DSS-UC and DSS-UC+Emapunil groups over the 9-day experimental period (n = 6 per group). *P < 0.05, **P < 0.01, ***P < 0.001, ns: not significant. Data are presented as mean ± SD. Statistical analyses were conducted using two-way ANOVA followed by Tukey’s *post-hoc* test. **(D)** Hematochezia in mice from the CON, DSS-UC and DSS-UC+Emapunil groups. **(E)** Abdominal cavity in mice from the CON, DSS-UC and DSS-UC+Emapunil groups. **(F)** DAI scores of the CON, DSS-UC and DSS-UC+Emapunil groups over the 9-day experimental period (n = 6 per group). *P < 0.05, **P < 0.01, ***P < 0.001, ns: not significant. Data are presented as mean ± SD. Statistical analyses were conducted using two-way ANOVA followed by Tukey’s *post-hoc* test. **(G)** Colon tissues isolated from mice in the CON, DSS-UC and DSS-UC+Emapunil groups. **(H)** Colon length in mice from the CON, DSS-UC and DSS-UC+Emapunil groups (n = 6 per group). *P < 0.05, **P < 0.01, ***P < 0.001, ns, not significant. Data are presented as mean ± SD. Statistical analyses were conducted using one-way ANOVA followed by Tukey’s *post-hoc* test.

Male wild-type C57BL/6 mice (age = 6–8 weeks) were purchased from the Laboratory Animal Centre of Southern Medical University (Guangzhou, China) and housed under specific pathogen-free conditions. The animals were maintained in a laboratory with constant temperature and humidity (21 °C ± 2 °C, 50% ± 5% humidity) under a 12-hour light/dark cycle. All the animals were acclimatized in the laboratory animal facility for 10 days prior to the start of experiments. The mice were randomly assigned to three groups (n = 6 per group): control, dextran sodium sulfate (DSS)-UC and DSS-UC+Emapunil. The control group received normal drinking water. In both the DSS-UC and DSS-UC+Emapunil groups, UC was induced by administering 3% DSS in drinking water for 7 consecutive days. The DSS-UC+Emapunil group also received concurrent intraperitoneal injections of 0.3 mg/kg Emapunil daily for 7 days, the control group and the DSS-UC groups were injected with the equal volume of solvent. Following DSS and/or Emapunil treatment, the mice were provided with regular drinking water for an additional 2 days. During disease progression in DSS-induced colitis model mice, body weight loss, stool consistency and faecal occult blood were recorded daily and used to calculate the disease activity index (DAI) (27). On day 9, the animals were euthanised via cervical dislocation and their serum and colorectal tissues were collected. The specific grouping is presented in **Figure 1**. The DSS-induced colitis model was established following the methodology described in our previous study (28). The effective concentration of Emapunil was determined based on preliminary investigations (24, 25). All animal experiments were approved by the Animal Protection and Use Committee of Southern Medical University. All animal models and experimental procedures were conducted in accordance with the Guide for the Care and Use of Laboratory Animals issued by the National Institutes of Health (Bethesda, MD, USA).

### Cell culture

Caco2 cells were cultured in Dulbecco’s modified Eagle’s medium (Gibco) containing 4.5 g/L glucose. THP-1 human monocytic leukaemia cells were maintained in RPMI-1640 medium. All culture media were supplemented with 10% foetal bovine serum, 100 U/mL penicillin and 100 μg/mL streptomycin. The cells were incubated under standard conditions: 37 °C with 5% CO_2_ and 95% humidity.

THP−1 cells were first differentiated into mature macrophages by treatment with 50 ng/mL Phorbol myristate acetate (PMA, MCE, HY-18739) for 24 hours (25). The differentiated macrophages were then treated with 500 ng/mL LPS (MCE, HY−D1056) for 24 hours to establish an M1−polarized macrophage model, followed by treatment with 600 ng/mL Emapunil for 24 hours. The cells were then harvested for RNA or protein analysis.

To achieve overexpression of ZBP1, THP-1 cells were transfected with a vector and ZBP1 plasmid (oneshine, G40021) using LipoRNAi™ Transfection Reagent (Beyotime, C0535). The culture medium was replaced with fresh medium 6 hours after transfection and the cells were collected at 48 hours post-transfection for RNA and protein analysis.

Human ZBP1 or TSPO mRNA expression were inhibited using small interfering RNA (siRNA). According to the manufacturer’s protocol, THP-1 cells were transfected for 24 h with 75 nM of either ZBP1-targeting siRNA (si-ZBP1 or TSPO) or negative control siRNA (si-NC) (GenePharma, Suzhou, China) using Lipofectamine 3000 (3 μL/mL). The cells were subsequently processed with TRIzol for RNA analysis. The sequence of ZBP1 siRNA is listed below.

Si-NC: forward: 5′-UUC UCC GAA CGU GUC ACG UTT-3′, reverse: 5′-ACG UGA CAC GUU CGG AGA ATT-3′;
Si-ZBP1: forward: 5′- GCU CCA ACA AGU GCA GCU UTT -3′, reverse: 5′- AAG CUG CAC UUG UUG GAG CTT -3′;
Si-TSPO: forward: 5′- CCA CAC UCA ACU ACU GCG UTT -3′, reverse: 5′- UAC GCA GUA GUU GAG UGU CTT -3′.

### Co-culture and protein extraction

Caco2 and THP-1 cells were seeded in the upper and lower chambers, respectively, of a 12-well Transwell plate at a density of 60%–70%. After treatment according to the indicated experimental group, the medium was discarded and the cells were gently rinsed with phosphate-buffered saline (PBS). After co-culture for 24 h, Caco2 cells were collected for protein extraction. Total protein was extracted from the Caco2 cells in each group using ice-cold RIPA lysis buffer. The protein concentration was determined using the BCA method and the protein samples were stored at −80 °C.

### Histological analysis

Thin sections of mouse tissue embedded in paraffin were cut to a thickness of 3 µm and subsequently dried in a 65 °C oven for 120 minutes. The sections were then routinely stained with haematoxylin and eosin (H&E) and periodic acid–Schiff (PAS). Following staining, the sections were dried again and mounted with neutral balsam. Finally, all the prepared slides were systematically examined and evaluated by experienced pathologists.

### Immunofluorescence staining

Tissue sections were baked at 65 °C for 2 hours, then subjected to sequential deparaffinisation and dehydration. The sections were rinsed in deionised water for 5 minutes. For antigen retrieval, the sections were incubated overnight in sodium citrate buffer maintained at 60 °C using a water bath.

The sections were incubated with Alexa Fluor 488- or Alexa Fluor 594-conjugated secondary antibodies (Life Technologies, Carlsbad, CA, USA) at room temperature in the dark for 1 hour. The nuclei were counterstained with 4′,6-diamidino-2-phenylindole (DAPI; Thermo Fisher Scientific, Waltham, MA, USA).

The following primary antibodies were used: mouse anti-CD68 (1:3000 dilution; proteintech, 66231-2-Ig), rabbit anti-ZBP1 (1:100 dilution; proteintech, 13285-1-AP), rabbit anti-occludin (1:100 dilution; Affinity, DF7504), rabbit anti-zona occludens-1 (ZO-1; 1:100 dilution; Affinity, AF5145), mouse anti-F4/80 (1:100 dilution; Santa Cruz, sc-377009), rabbit anti-GSDMD (1:100 dilution for IF; proteintech, 20770-1-AP) and rabbit anti-iNOS (1:100 dilution for IF; proteintech, 18985-1-AP).

### Cell IF staining

During co-culture, Caco2 cells were seeded onto 20-mm round coverslips. After the co-culture period, the cells were fixed with 4% paraformaldehyde for 15 minutes. After washing with PBS, the cells were blocked with 10% goat serum at 37 °C for 1 hour and then incubated overnight at 4 °C with primary antibodies diluted in PBS containing 1% bovine serum albumin and 0.1% Triton X-100. Subsequently, the cells were incubated with Alexa Fluor 488- or Alexa Fluor 594-conjugated secondary antibodies (Life Technologies, Carlsbad, CA, USA) for 1 hour at room temperature in the dark. The nuclei were counterstained with 4′,6-diamidino-2-phenylindole (DAPI; Thermo Fisher Scientific, Waltham, MA, USA). After additional PBS washes, DAPI staining solution was added to each well and the images were acquired using an inverted fluorescence microscope under corresponding fluorescence channels. The primary antibodies used in this study were as follows: occludin (1:100 dilution; Affinity, DF7504) and rabbit anti-phosphorylated ZO-1 (1:100 dilution; Affinity, AF5145).

### Quantitative reverse transcription-polymerase chain reaction (RT-qPCR)

Total RNA was extracted from tissues or cells using TRIzol in accordance with the manufacturer’s instructions and the obtained RNA samples were stored at -80 °C. The RNA concentrations of the samples were measured and the samples were appropriately diluted to ensure uniformity across the reactions. Reverse transcription was performed in a 20-µL reaction volume, using no more than 1000 ng of total RNA to synthesise cDNA. The resulting cDNA served as the template for real-time quantitative PCR amplification. The amplification results were analysed using the ABI 7300 real-time fluorescence quantitative PCR system and the Ct values were recorded. GAPDH was used as the internal reference gene and the relative expression levels of the target genes were calculated using the 2^-ΔΔCt^ method. The primer sequences used are listed below.

ZBP1: forward primer 5’- TGGTCATCCCAAGCACTG-3’, reverse primer 5’- GGCGGTAAATCGTCCATGCT-3’;
IL-1β: forward primer 5’-ATGATGGCTTATTACAGTGGCAA-3’, reverse primer 5’- GTCGGAGATTCGTAGCTGGA-3’;
IL-6: forward primer 5’-ACTCACCTCTTCAGAACGAATTG-3’, reverse primer 5’- CCATCTTTGGAAGGTTCAGGTTG-3’;
CD80: forward primer 5’-AAACTCGCATCTACTGGCAAA-3’, reverse primer 5’- GGTTCTTGTACTCGGGCCATA-3’;

### Enzyme-linked immunosorbent assay

Human IL-1β and IL-6 ELISA kits (Elabscience Biotechnology, Bethesda, MD, USA; E-EL-H0149 and E-EL-H6156) were used to measure the concentrations of IL-1β and IL-6 in the culture supernatants of THP-1 cells from each experimental group. Mouse IL-1β and IL-6 ELISA kits (Elabscience Biotechnology, Bethesda, MD, USA; E-EL-M0044c and E-EL-M0049c) were used to determine the concentrations of IL-1β and IL-6 in serum from each group of mice.

### Western blot analysis

Caco2 cells were lysed in 100 µL of RIPA buffer (Beyotime Institute of Biotechnology, Jiangsu, China) supplemented with protease and phosphatase inhibitors. Protein aliquots (10 µg) were separated by 10% SDS-polyacrylamide gel electrophoresis and subsequently transferred onto PVDF membranes. The membranes were blocked with 5% skim milk and then incubated with primary antibodies. Immunoreactive bands were visualised using an enhanced chemiluminescence kit (Santa Cruz).The primary antibodies used for western blotting were as follows: rabbit anti-GAPDH (1:10,000 dilution; Abcam, ab181603), rabbit anti-occludin (1:1,000 dilution; Affinity, DF7504), rabbit anti-ZO-1 (1:1,000 dilution; Affinity, AF5145), rabbit anti-IKBA (1:1,000 dilution; Abmart, TA2002), rabbit anti-ERK (1:1,000 dilution; Abmart, T40071) and rabbit anti-phosphorylated ERK (1:1,000 dilution; Abmart, TA1015), rabbit anti-ZBP1 (1:1000 dilution; proteintech, 13285-1-AP), rabbit anti-NLRP3 (1:1000 dilution; proteintech, 30109-1-AP), rabbit anti-cleaved N-terminal GSDMD (1:1,000 dilution; Abcam, EPR20829-408), rabbit anti-Caspase1-p20 (1:5000 dilution; proteintech, 22915-1-AP).

### mRNA transcriptome sequencing

The mRNA transcriptome sequencing data were derived from our previously published study (25). Total RNA was extracted from RAW264.7 cells treated with 500 ng/mL LPS alone or 500 ng/mL LPS combined with 600 ng/mL Emapunil using TRIZOL reagent (n=3 per group). Subsequent procedures, including RNA−seq library construction, high−throughput sequencing, sequence alignment, differential gene screening, and signaling pathway enrichment analysis, were performed by Shanghai Liebing Biopharmaceutical Technology Co., Ltd. (NovelBio).

### Statistical analysis

Data were compiled and tabulated using Microsoft Excel 2019. Images were processed using Adobe Photoshop 2020 and statistical analyses were performed using GraphPad Prism 8.0.2. The specific statistical methods applied to each set of results are described in the corresponding figure legends. Statistical significance was defined as a P value < 0.05 and all experiments were repeated independently two to three times.

## Results

### ZBP1 is upregulated in UC colorectal macrophages

**Figure 2:**
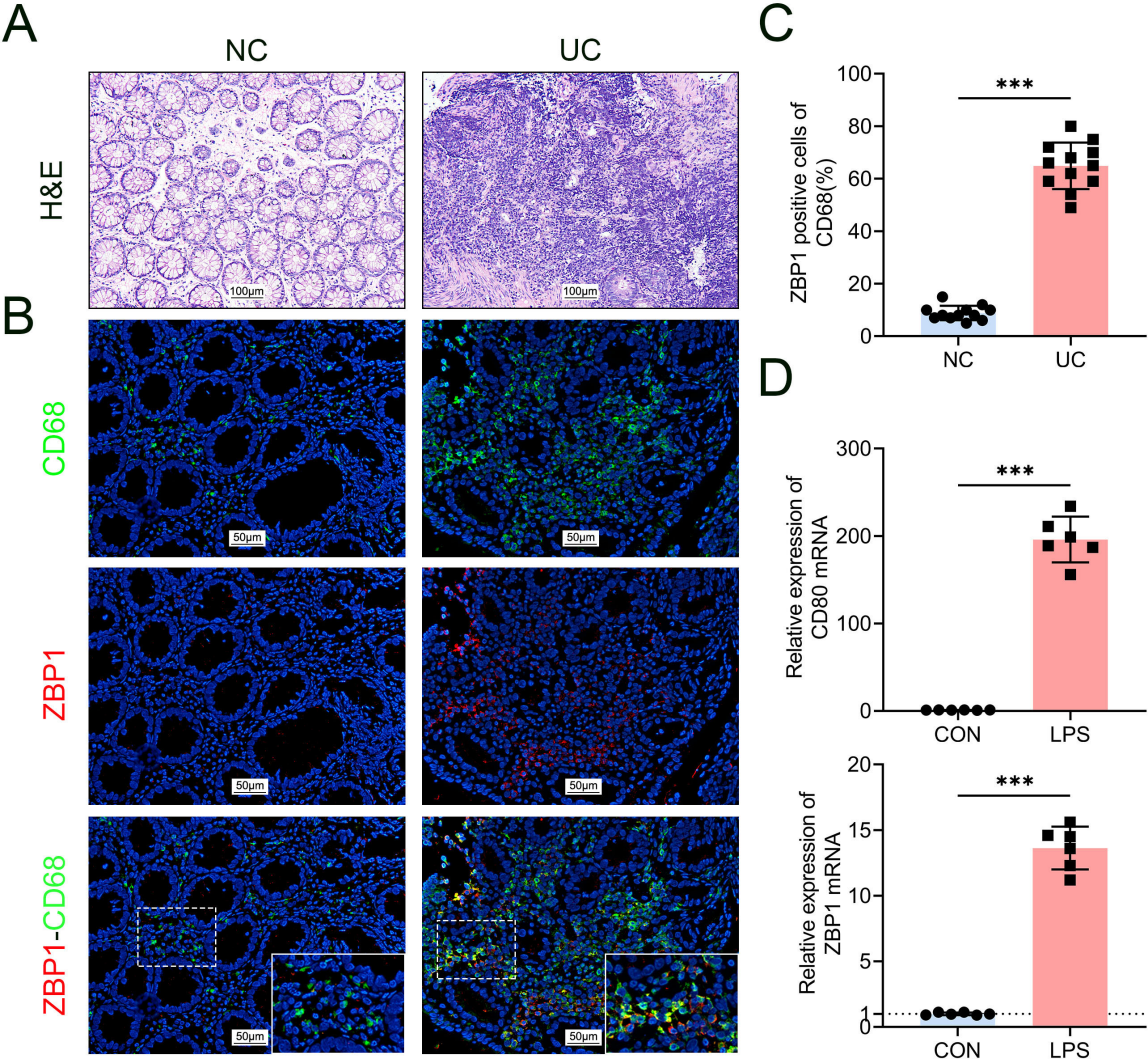
ZBP1 is upregulated in colorectal macrophages from UC patients and in LPS-stimulated M1-polarized macrophages. **(A)** H&E staining of colorectal tissues from NC and UC patients. Scale bar: 100 μm. **(B)** IF staining of CD68 (green), ZBP1 (red) and merged images (ZBP1-CD68 co-localization) in colorectal tissues from NC and UC patients. Scale bar: 50 μm. Insets show magnified views of the boxed regions. **(C)** Quantification of ZBP1-positive cells among CD68-positive macrophages in colorectal tissues from NC and UC patients (n = 12 per group). *P < 0.05, **P < 0.01, ***P < 0.001, ns: not significant. Data are presented as mean ± SD. Statistical analyses were conducted using an unpaired two−tailed Student’s t−test. **(D)** Relative mRNA expression of CD80 and ZBP1 in THP-1 cells treated with or without LPS (n = 6 per group). *P < 0.05, **P < 0.01, ***P < 0.001, ns, not significant. Data are presented as mean ± SD. Statistical analyses were conducted using an unpaired two−tailed Student’s t−test.

To investigate the expression of ZBP1 during the onset and progression of UC, colorectal tissue samples from patients with UC and normal controls (NC) were collected (**Figure 2A**). Significant upregulation of ZBP1 expression was detected in colorectal tissues from patients with UC by IF analysis (**Figure 2B**). Co-localization analysis of ZBP1 and the macrophage marker CD68 revealed that the increase in ZBP1 expression was more pronounced in macrophages than in epithelial cells (**Figures 2B, C**).

THP−1 cells were first differentiated into mature macrophages by treatment with 50 ng/mL PMA for 24 hours (**Supplementary Figure 1**). Then an inflammatory environment was simulated via LPS stimulation of THP-1 cells *in vitro*. The qPCR results demonstrated that LPS stimulation led to a significant increase in the expression of CD80, an M1 polarization-related marker, indicating that THP-1 cells underwent M1 polarization (**Figure 2D**). In these M1-polarized macrophages, ZBP1 expression was significantly upregulated (**Figure 2D**).

### ZBP1 induces macrophage pyroptosis and promotes polarization toward the M1 phenotype, activating the NF-κB pathway

**Figure 3:**
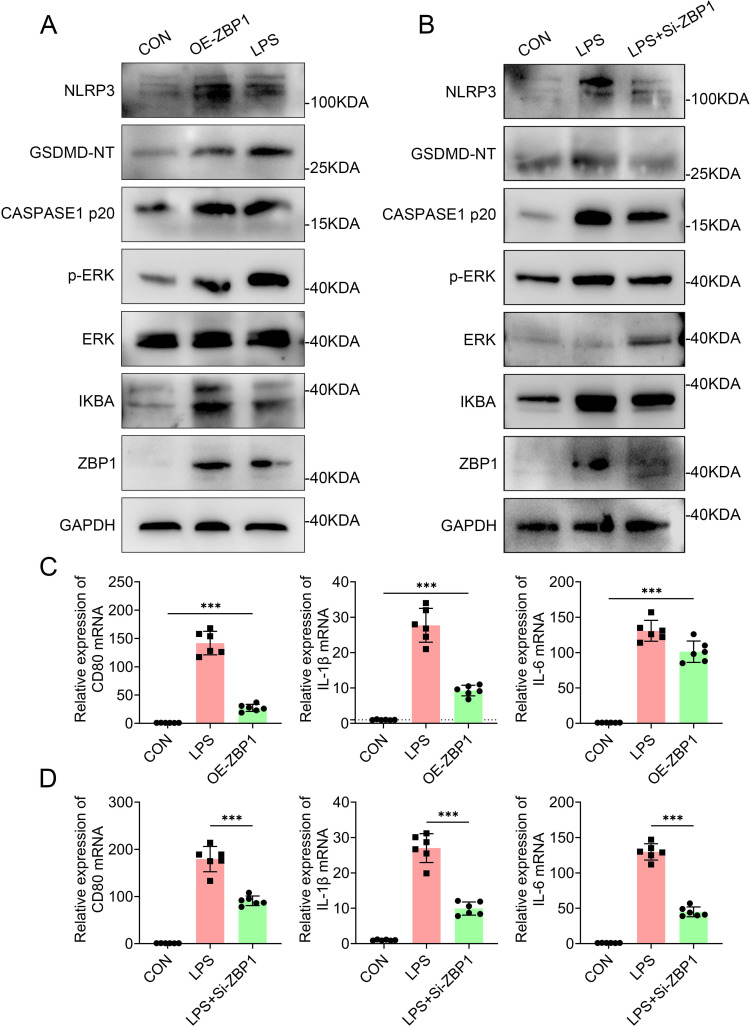
ZBP1 induces pyroptosis and M1 polarization in macrophages, activating the NF-κB pathway. **(A)** WB analysis of NLRP3, GSDMD-NT, Caspase1 p20, p-ERK, ERK, IKBA and ZBP1 in THP-1 cells under control, OE-ZBP1 and LPS stimulation conditions. **(B)** Western blot analysis of NLRP3, GSDMD-NT, Caspase1 p20, p-ERK, ERK, IKBA and ZBP1 in THP-1 cells under control, LPS stimulation and LPS stimulation with ZBP1 knockdown conditions. **(C)** Relative mRNA expression of CD80, IL-1β and IL-6 in THP-1 cells under control, LPS and OE-ZBP1 stimulation conditions (n = 6 per group). *P < 0.05, **P < 0.01, ***P < 0.001, ns: not significant. Data are presented as mean ± SD. Statistical analyses were conducted using one-way ANOVA followed by Tukey’s post-hoc test. **(D)** Relative mRNA expression of CD80, IL-1β and IL-6 in THP-1 cells under control, LPS stimulation and LPS stimulation with ZBP1 knockdown conditions (n = 6 per group). *P < 0.05, **P < 0.01, ***P < 0.001, ns, not significant. Data are presented as mean ± SD. Statistical analyses were conducted using one-way ANOVA followed by Tukey’s post-hoc test.

Next, ZBP1 was overexpressed in THP-1 cells (OE-ZBP1) to elucidate the functional consequences of ZBP1 upregulation in macrophages. WB analysis indicated that ZBP1 overexpression triggered pyroptosis in macrophages, as evidenced by the upregulated levels of NLRP3, GSDMD-NT and Caspase1 p20; concurrently, ZBP1 overexpression enhanced activation of the NF-κB signaling pathway, which manifested as increased p-ERK and IKBA (**Figure 3A**). The RT-qPCR results demonstrated that ZBP1 overexpression significantly increased the levels of mRNAs encoding CD80, IL-1β and IL-6 (**Figure 3C**), suggesting that high expression of ZBP1 induces macrophage polarization towards the M1 phenotype.

Conversely, siRNA-mediated knockdown of ZBP1 (si-ZBP1) in LPS-stimulated THP-1 cells effectively suppressed the expression of pyroptosis-related proteins including NLRP3, GSDMD-NT and Caspase1 p20 and attenuated activation of the NF-κB pathway (**Figure 3B**). It also inhibited the M1 polarization phenotype and reduced the transcription of pro-inflammatory cytokines including IL-1β and IL-6 (**Figure 3D**).

### ZBP1-overexpressing macrophages disrupt intestinal epithelial barrier integrity

**Figure 4:**
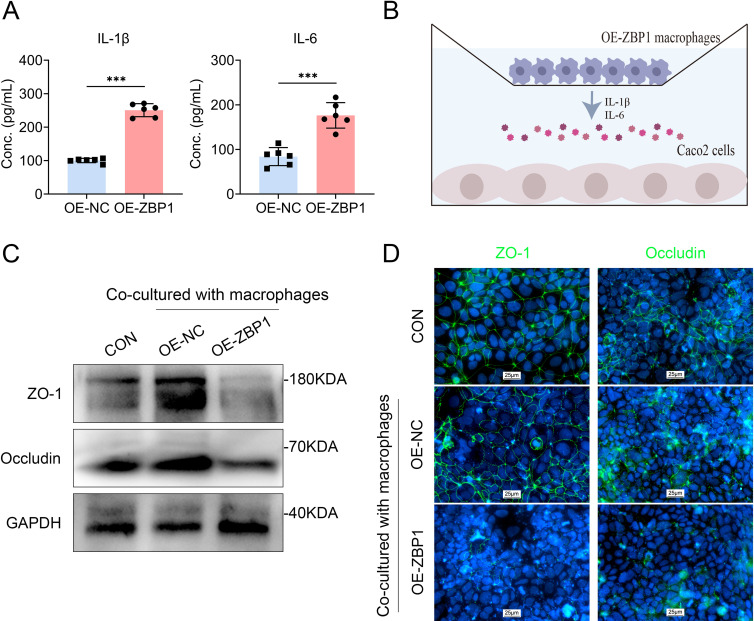
ZBP1-overexpressing macrophages disrupt intestinal epithelial barrier integrity. **(A)** Concentrations of IL-1β and IL-6 in the culture supernatants of THP-1 cells with OE-ZBP1 or control (n = 6 per group). *P < 0.05, **P < 0.01, ***P < 0.001, ns: not significant. Data are presented as mean ± SD. Statistical analyses were conducted using an unpaired two−tailed Student’s t−test. **(B)** Schematic diagram of the Transwell co-culture model, in which THP-1 cells are seeded in the upper chamber and Caco2 intestinal epithelial cells are seeded in the lower chamber. **(C)** WB analysis of ZO-1 and occludin in Caco2 cells co-cultured with control macrophages or OE-ZBP1 macrophages. **(D)** Cell IF staining of ZO-1 and occludin in Caco2 cells under control conditions or co-cultured with control macrophages or OE-ZBP1 macrophages. Scale bar: 25 μm.

To investigate how macrophages with high ZBP1 expression affect the intestinal epithelial barrier, we first detected the pro-inflammatory cytokine secretion of ZBP1-overexpressing THP-1 cells. ELISA results showed that the concentrations of IL-1β and IL-6 in the culture supernatants of OE-ZBP1 THP-1 cells were significantly increased compared with the control group (**Figure 4A**).

We then established a transwell co-culture model with THP-1 cells in the upper chamber and Caco2 cells in the lower chamber (**Figure 4B**). WB analysis revealed that, compared with the OE-NC group, the levels of tight junction proteins ZO-1 and occludin in Caco2 cells were significantly reduced following co-culture with ZBP1-overexpressing THP-1 cells (**Figure 4C**). IF staining further confirmed that the expression and distribution of ZO-1 and occludin in Caco2 cells were disrupted after co-culture with OE-ZBP1 macrophages (**Figure 4D**), indicating impaired intestinal epithelial barrier integrity.

### Emapunil suppresses ZBP1-mediated pyroptosis and pro-inflammatory responses in macrophages

**Figure 5:**
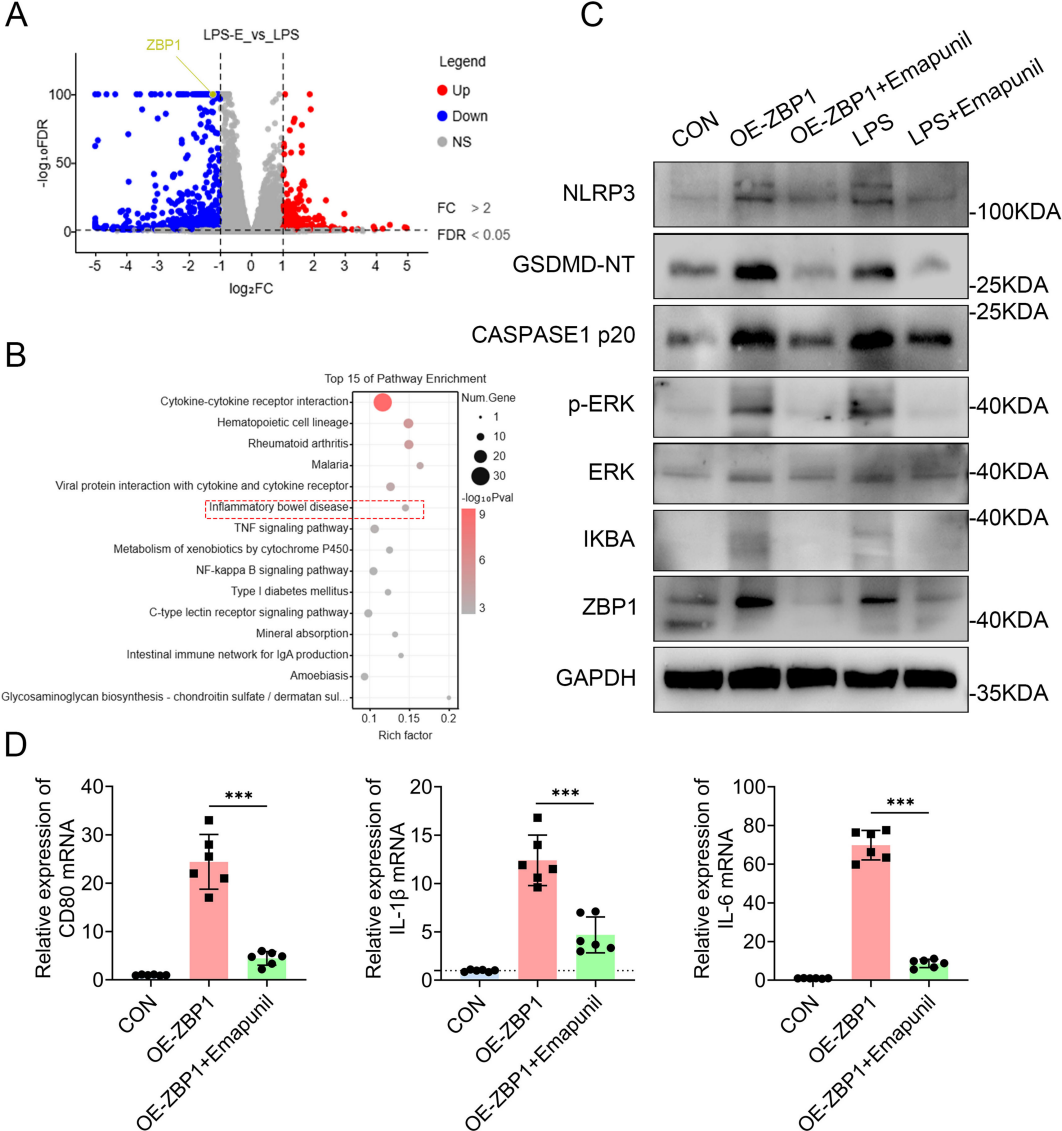
Emapunil suppresses ZBP1-mediated pyroptosis and pro-inflammatory responses in macrophages. **(A)** Volcano plot of differentially expressed genes in LPS-stimulated RAW264.7 cells treated with Emapunil compared with LPS treatment. Red: upregulated genes (log_2_FC > 1, FDR < 0.05); blue: downregulated genes (log_2_FC < -1, FDR < 0.05); gray: not significant (NS). **(B)** Top 15 KEGG pathway enrichment analysis of differentially expressed genes. **(C)** WB analysis of NLRP3, GSDMD-NT, Caspase1 p20, p-ERK, ERK, IKBA and ZBP1 in THP-1 cells under control, OE-ZBP1, OE-ZBP1 with Emapunil treatment, LPS stimulation and LPS with Emapunil treatment conditions. **(D)** Relative mRNA expression of CD80, IL-1β and IL-6 in THP-1 cells under control, OE-ZBP1 and OE-ZBP1 with Emapunil treatment conditions (n = 6 per group). *P < 0.05, **P < 0.01, ***P < 0.001, ns, not significant. Data are presented as mean ± SD. Statistical analyses were conducted using one-way ANOVA followed by Tukey’s *post-hoc* test.

We reanalyzed RNA-seq data (25) from LPS-stimulated RAW264.7 cells treated with Emapunil and confirmed that the gene encoding ZBP1 was significantly downregulated (**Figure 5A**, **Supplementary Table 2**), suggesting that Emapunil could downregulate ZBP1 expression. Kyoto Encyclopedia of Genes and Genomes (KEGG) pathway enrichment analysis of the differentially expressed genes revealed significant enrichment of IBD-related pathways (**Figure 5B**, **Supplementary Table 3**), indicating potential links among Emapunil, ZBP1 and IBD.

We confirmed that Emapunil exhibited no cytotoxicity in THP-1 or Caco2 cells within the concentration range of 0–600 ng/mL (**Supplementary Figures 2A, B**), establishing this as a safe concentration range. Within this range, the inhibitory effect of Emapunil on macrophage M1 polarization increased in a concentration-dependent manner, being most pronounced at 600 ng/mL, and this inhibitory effect also increased with prolonged stimulation time (**Supplementary Figures 2C, D**). Specifically, Emapunil treatment significantly suppressed the upregulation of ZBP1 protein levels induced by LPS or ZBP1 overexpression in THP-1 cells, while concomitantly attenuating the expression of pyroptosis-related proteins (NLRP3, GSDMD-NT, Caspase1 p20) and the activation of the NF-κB pathway (**Figure 5C**). Moreover, Emapunil treatment reduced the transcription of the M1 polarization marker CD80 and the pro-inflammatory cytokines IL-1β and IL-6 in ZBP1-overexpressing THP-1 cells (**Figure 5D**), indicating that its anti-inflammatory effect depends on downregulating ZBP1.

### Emapunil reverses ZBP1-overexpressing macrophage-induced intestinal epithelial barrier damage

**Figure 6:**
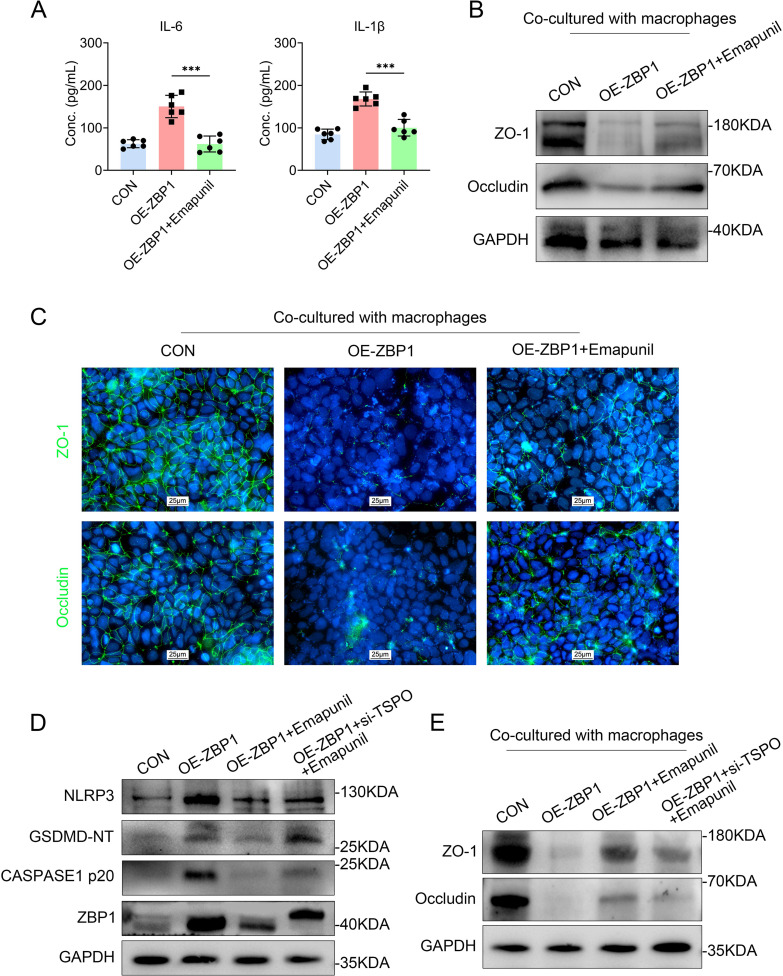
Emapunil reverses ZBP1-overexpressing macrophage-induced intestinal epithelial barrier damage. **(A)** Concentrations of IL-6 and IL-1β in the culture supernatants of THP-1 cells under control, OE-ZBP1 and OE-ZBP1 with Emapunil treatment conditions (n = 6 per group). *P < 0.05, **P < 0.01, ***P < 0.001, ns: not significant. Data are presented as mean ± SD. Statistical analyses were conducted using one-way ANOVA followed by Tukey’s *post-hoc* test. **(B)** WB analysis of ZO-1 and occludin in Caco2 cells co-cultured with control, OE-ZBP1 THP-1 cells or OE-ZBP1 THP-1 cells treated with Emapunil. **(C)** Cell IF staining of ZO-1 and occludin in Caco2 cells co-cultured with control, OE-ZBP1 THP-1 cells or OE-ZBP1 THP-1 cells treated with Emapunil. Scale bar: 25 μm. **(D)** WB analysis of NLRP3, GSDMD-NT, Caspase1 p20 and ZBP1 in THP-1 cells under control, OE-ZBP1, OE-ZBP1 with Emapunil treatment, OE-ZBP1 and si-TSPO with Emapunil treatment conditions. **(E)** WB analysis of ZO-1 and occludin in Caco2 cells co-cultured with control, OE-ZBP1 THP-1 cells, OE-ZBP1 THP-1 cells treated with Emapunil or OE-ZBP1 and si-TSPO THP-1 cells treated with Emapunil.

We first detected the effect of Emapunil on pro-inflammatory cytokine secretion in ZBP1-overexpressing THP-1 cells. ELISA results showed that the concentrations of IL-1β and IL-6 in the culture supernatants of OE-ZBP1 THP-1 cells were significantly increased, whereas Emapunil treatment reduced the levels of these pro-inflammatory cytokines (**Figure 6A**).

We then used the transwell co-culture model to evaluate the protective effect of Emapunil on the intestinal epithelial barrier. WB analysis revealed that Emapunil treatment significantly increased the expression of tight junction proteins ZO-1 and occludin in Caco2 cells co-cultured with ZBP1-overexpressing THP-1 cells (**Figure 6B**). IF staining further confirmed that Emapunil alleviated the disruption of ZO-1 and occludin expression and distribution in Caco2 cells induced by OE-ZBP1 macrophages (**Figure 6C**).

Since TSPO is a selective ligand of Emapunil, we knocked down TSPO in THP-1 cells to determine whether the effect of Emapunil depends on TSPO. The results showed that TSPO knockdown largely abolished the suppressive effects of Emapunil on ZBP1 expression and pyroptosis, as evidenced by the restored expression of pyroptosis−related proteins (NLRP3, GSDMD−NT, Caspase1 p20) (**Figure 6D**). In the co−culture system, TSPO knockdown also attenuated the protective effect of Emapunil on tight junction proteins, manifested as decreased expression of ZO−1 and occludin (**Figure 6E**). These findings indicate that Emapunil downregulates ZBP1 in a TSPO−dependent manner.

### Emapunil ameliorates DSS-induced colitis in mice

The therapeutic potential of Emapunil was evaluated in a DSS-induced colitis mouse model, with mice randomly divided into three groups: control (CON), DSS-UC and DSS-UC+Emapunil (**Figure 1A**). Emapunil treatment significantly alleviated the body weight loss of mice induced by DSS over the 9-day experimental period (**Figures 1B, C**). Hematochezia symptoms in DSS-UC mice were also improved by Emapunil treatment (**Figure 1D**). Notably, DSS caused significant colorectal distension in mice, while Emapunil treatment alleviated the pathological changes in the abdominal cavity of DSS-induced colitis mice (**Figure 1E**).

In addition, Emapunil treatment significantly reduced the DAI scores of DSS-UC mice during the experimental period (**Figure 1F**) and mitigated the colon length shortening induced by DSS (**Figures 1G, H**).

### Emapunil ameliorates colorectal tissue damage and preserves intestinal barrier integrity in DSS-induced colitis mice

**Figure 7:**
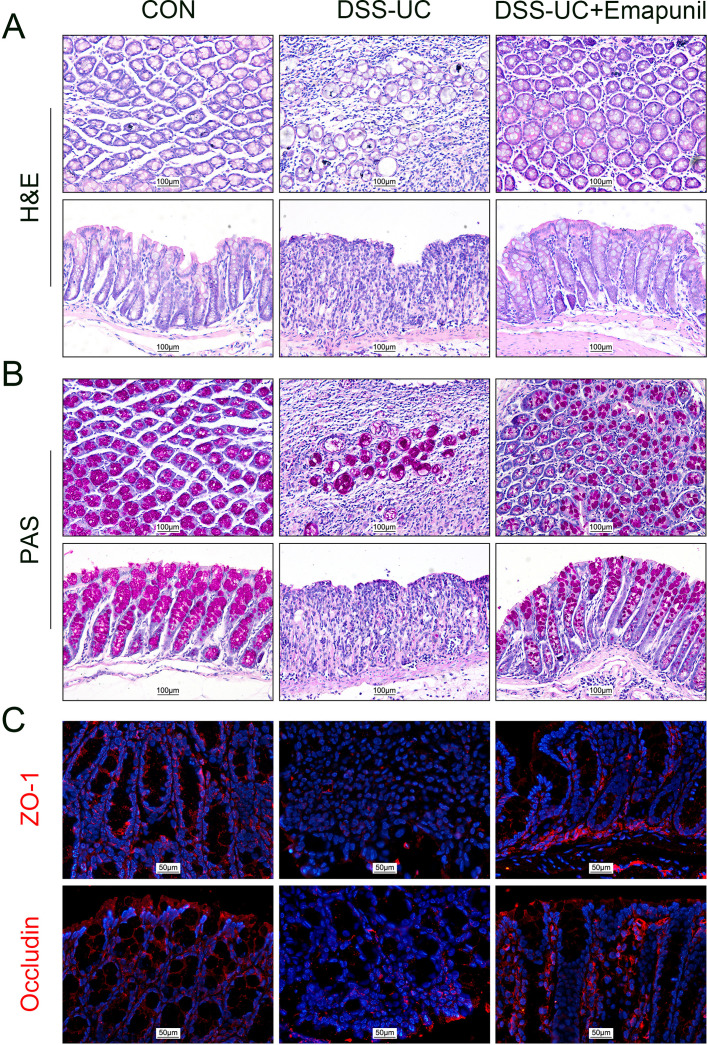
Emapunil ameliorates colorectal tissue damage and preserves intestinal barrier integrity in DSS-induced colitis mice. **(A)** H&E staining of colorectal tissues from CON, DSS-UC and DSS-UC+Emapunil mice. Scale bar: 100 μm. **(B)** PAS staining of colorectal tissues from CON, DSS-UC and DSS-UC+Emapunil mice. Scale bar: 100 μm. **(C)** Cell IF staining of tight junction proteins ZO-1 and occludin in colorectal tissues from CON, DSS-UC and DSS-UC+Emapunil mice. Scale bar: 50 μm.

H&E staining showed that DSS stimulation caused severe colorectal tissue damage including inflammatory cell infiltration, mucosal ulceration and crypt destruction in mice, while Emapunil treatment markedly ameliorated these pathological changes (**Figure 7A**). PAS staining revealed that DSS-induced colitis led to a significant reduction in colonic mucosal goblet cells and Emapunil treatment alleviated this loss of goblet cells and preserved the mucosal structure of colorectal tissues (**Figure 7B**).

IF staining of colorectal tissues revealed that the expression of tight junction proteins ZO-1 and occludin was significantly reduced and disrupted in DSS-UC mice, while Emapunil treatment effectively preserved the expression and normal distribution of ZO-1 and occludin (**Figure 7C**), indicating that Emapunil protects intestinal epithelial barrier integrity in DSS-induced colitis mice.

### Emapunil inhibits ZBP1-mediated pyroptosis and M1 polarization in colorectal macrophages of DSS-induced colitis mice

**Figure 8:**
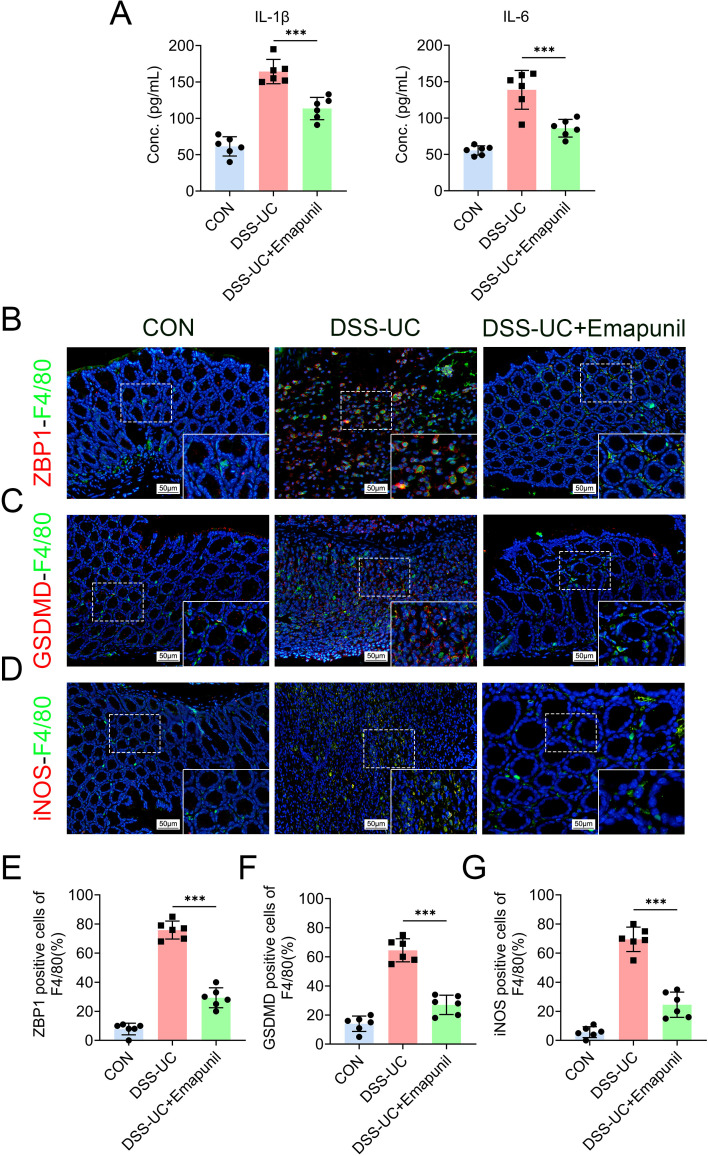
Emapunil inhibits ZBP1-mediated pyroptosis and M1 polarization in colonic macrophages of DSS-induced colitis mice. **(A)** Concentrations of IL-1β and IL-6 in the serum of control, DSS-UC and DSS-UC+Emapunil mice (n = 6 per group). *P < 0.05, **P < 0.01, ***P < 0.001, ns: not significant. Data are presented as mean ± SD. Statistical analyses were conducted using one-way ANOVA followed by Tukey’s *post-hoc* test. **(B)** IF co-staining of F4/80 (green) and ZBP1 (red) in colorectal tissues from CON, DSS-UC, and DSS-UC+Emapunil mice. Scale bar: 50 μm. **(C)** IF co-staining of F4/80 (green) and GSDMD (red) in colorectal tissues from CON, DSS-UC, and DSS-UC+Emapunil mice. Scale bar: 50 μm. **(D)** IF co-staining of F4/80 (green) and iNOS (red) in colorectal tissues from CON, DSS-UC, and DSS-UC+Emapunil mice. Scale bar: 50 μm. **(E)** Quantification of ZBP1-positive cells among F4/80-positive macrophages in colorectal tissues from CON, DSS-UC, and DSS-UC+Emapunil mice (n = 6 per group). *P < 0.05, **P < 0.01, ***P < 0.001, ns: not significant. Data are presented as mean ± SD. Statistical analyses were conducted using one-way ANOVA followed by Tukey’s *post-hoc* test. **(F)** Quantification of GSDMD-positive cells among F4/80-positive macrophages in colorectal tissues from CON, DSS-UC, and DSS-UC+Emapunil mice (n = 6 per group). *P < 0.05, **P < 0.01, ***P < 0.001, ns: not significant. Data are presented as mean ± SD. Statistical analyses were conducted using one-way ANOVA followed by Tukey’s *post-hoc* test. **(G)** Quantification of iNOS-positive cells among F4/80-positive macrophages in colorectal tissues from CON, DSS-UC, and DSS-UC+Emapunil mice (n = 6 per group). *P < 0.05, **P < 0.01, ***P < 0.001, ns, not significant. Data are presented as mean ± SD. Statistical analyses were conducted using one-way ANOVA followed by Tukey’s *post-hoc* test.

Consistent with the *in vitro* results, the systemic inflammatory response in DSS-induced colitis mice was significantly elevated, as evidenced by the increased concentrations of pro-inflammatory cytokines IL-1β and IL-6 in serum; however, Emapunil treatment effectively reduced the serum levels of these cytokines (**Figure 8A**).

IF co-staining and quantitative analysis clearly confirmed the pathological alterations in colonic macrophages induced by DSS stimulation: specifically, DSS modeling significantly enhanced ZBP1 expression in colorectal macrophages (**Figures 8B, E**) and concurrently promoted the polarization of macrophages toward the M1 phenotype, which was reflected by a marked increase in the co-localization of F4/80 with the M1 polarization marker iNOS (**Figures 8D, G**). In contrast, Emapunil treatment significantly reversed both of these changes (**Figures 8B, D, E, G**).

Furthermore, Emapunil treatment suppressed the expression of GSDMD in macrophages (**Figures 8C, F**), suggesting that the inhibition of ZBP1 by Emapunil is accompanied by the suppression of pyroptosis.

## Discussion

In this study, we investigated the role of ZBP1-dependent macrophage pyroptosis and imbalanced polarization in inflammation associated with UC. Specifically, we demonstrated that Emapunil alleviates colorectal inflammation and protects the epithelial barrier by downregulating ZBP1 in macrophages, thereby inhibiting ZBP1-mediated macrophage pyroptosis and M1 polarization and subsequently suppressing the release of pro-inflammatory cytokines and activation of the NF-κB pathway.

Pyroptosis, a pro-inflammatory form of programmed cell death mediated by inflammasomes, has attracted increasing attention due to its role in the pathophysiology of UC. A hallmark of pyroptosis is the formation of pores on the plasma membrane by gasdermin family proteins such as GSDMD; this leads to cell rupture and the massive release of pro-inflammatory cytokines such as IL-1β and IL-18, which exacerbates tissue damage (2, 3). Studies have demonstrated that inhibiting GSDMD can alleviate UC progression (6, 29); however, the precise mechanisms by which pyroptosis contributes to UC pathogenesis remain unclear.

Recent studies have identified ZBP1 as a key upstream regulator of pyroptosis: ZBP1 senses damage signals to activate the NLRP3 inflammasome, which in turn induces Caspase1 cleavage into p20 and subsequent GSDMD cleavage to GSDMD-NT, ultimately driving pyroptotic cell death and inflammatory responses (10, 11, 30). ZBP1 has been shown to exert pro-inflammatory effects in various inflammatory models, including those of the liver, bile duct and lungs. In primary biliary cirrhosis and primary sclerosing cholangitis, ZBP1 interacts with NLRP3 to regulate cell death and inflammatory responses (31). In sepsis-induced acute lung injury, ZBP1 expression is upregulated in macrophages and induces pyroptosis triggered by NLRP3 inflammasome activation, which amplifies the inflammatory disease response and leads to endothelial dysfunction and cellular damage (32). Studies of UC have reported that elevated expression of ZBP1 in intestinal epithelial cells is closely associated with pyroptosis (15, 16). However, studies investigating ZBP1 in UC have been restricted exclusively to intestinal epithelial cells, and the expression and functional significance of ZBP1 in colorectal macrophages remain to be elucidated. In contrast, the present study systematically characterized the expression pattern and regulatory role of ZBP1 in colorectal macrophages during UC pathogenesis for the first time. Our findings confirm this elevated expression of ZBP1 in the colorectal tissues of patients with UC. Notably, our experiments revealed that, among the various cell types in colorectal tissues, high ZBP1 expression was particularly prominent in macrophages, which highlights the necessity to elucidate its specific regulatory role in this key immune effector cell type in UC pathogenesis.

Macrophages are classical immune cells with a crucial role in the occurrence and development of UC (17, 33). Macrophages can differentiate into pro-inflammatory M1 macrophages, which secrete large amounts of pro-inflammatory cytokines such as IL-1β, IL-6 and TNF-α and activate inflammatory signaling pathways, including NF-κB, thereby accelerating the progression of UC (34, 35). Given their significant role in UC, macrophages have been recognised as potential target cells in UC therapy. In this study, we revealed that ZBP1 is highly expressed in macrophages from patients with UC. Through *in vivo* and vitro experiments, we demonstrated that the elevated ZBP1 levels in both UC colorectal tissues and LPS-stimulated macrophages triggered pyroptosis and promoted polarization towards the M1 phenotype. This process was accompanied by the release of inflammatory factors and activation of the NF-κB signaling pathway, ultimately leading to disruption of the colorectal epithelial tight junction structure. Conversely, knockdown of ZBP1 effectively reversed the aforementioned pathological phenotypes induced by LPS, thus establishing macrophage ZBP1 as a crucial factor in the pathogenesis and progression of UC. These findings indicate that ZBP1 integrates two key pro-inflammatory pathways in macrophages, namely pyroptosis and polarization and suggest that targeting ZBP1 in macrophage may be a feasible therapeutic strategy for UC.

Previous transcriptomic analyses showed that Emapunil can inhibit LPS-induced ZBP1 expression in macrophages and attenuate their pro-inflammatory responses, while pathway enrichment analysis emphasised the relevance of ZBP1 in IBD-associated signaling (25). Based on these earlier findings, we further validated the therapeutic potential of Emapunil in both cellular and animal models. Previous studies have identified the anti-inflammatory and neuroprotective effects of Emapunil in central nervous system disorders (36, 37). In osteoarthritis, Emapunil was shown to alleviate inflammatory progression by inhibiting macrophage M1 polarization (25). We found that Emapunil significantly inhibited ZBP1 expression in macrophages under inflammatory conditions and reversed ZBP1 overexpression-induced macrophage pyroptosis and M1 polarization, thereby suppressing the release of pro-inflammatory cytokines and activation of the NF-κB pathway. In DSS-induced colitis model mice, Emapunil treatment not only reduced ZBP1 expression in colorectal tissue macrophages but also concurrently inhibited the expression of downstream pyroptosis-related proteins, including NLRP3, GSDMD-NT and Caspase1 p20. Furthermore, Emapunil suppressed M1 polarization in macrophages, ultimately ameliorating pathological intestinal damage and enhancing intestinal barrier integrity. These results extend the potential therapeutic application of Emapunil to UC for the first time and suggest that the drug’s mechanism of action may be associated with its regulation of ZBP1 in macrophages.

Emapunil is a selective ligand of TSPO (24). Consistent with the TSPO-dependent anti-inflammatory effects of Emapunil reported in previous studies (25), our TSPO knockout experiments revealed that Emapunil downregulates ZBP1 expression in a TSPO-dependent manner, as TSPO deficiency largely abolished the suppressive effects of Emapunil on ZBP1-mediated pyroptosis. These findings suggest that TSPO acts upstream of ZBP1 in mediating the anti-inflammatory action of Emapunil in macrophages. Nevertheless, the precise molecular link between TSPO and ZBP1 remains to be determined.

This study is the first to systematically characterize the expression pattern and regulatory role of ZBP1 in colorectal macrophages during UC progression and to demonstrate that Emapunil exerts anti−colorectal inflammation effects by targeting the ZBP1 pathway in a TSPO−dependent manner, providing new mechanistic insights and a potential therapeutic strategy for UC.

This study still has several limitations, which are specified as follows: First, although we confirmed via TSPO knockout cell experiments that Emapunil can downregulate ZBP1 expression in a TSPO-dependent manner, the specific molecular mechanism governing the regulatory relationship between TSPO and ZBP1 has not been fully elucidated and thus requires further investigation in subsequent experiments. Second, the dosage of Emapunil employed in *in vivo* experiments was determined with reference to relevant studies on other prior disease models, and no systematic dose-response relationship study was performed in the DSS-induced colitis model. Consequently, the optimal administration dose of Emapunil for the treatment of UC remains to be further optimized and validated. Third, the number of clinical samples included in this study was relatively small, which may compromise the generalizability of the research findings, and their reliability needs to be further confirmed in a larger cohort of clinical patients. Fourth, while we assessed the M1 polarization status of macrophages by detecting CD80 expression levels and pro-inflammatory cytokine secretion, we did not conduct relevant functional experiments such as those evaluating phagocytic function. Given that the phagocytic function of macrophages has garnered increasing attention in research on the pathogenesis of UC (38–40), Emapunil may modulate macrophage phagocytic function by regulating ZBP1, thereby exerting a therapeutic effect on UC; this potential mechanism merits in-depth exploration in future studies.

## Conclusion

**Figure 9:**
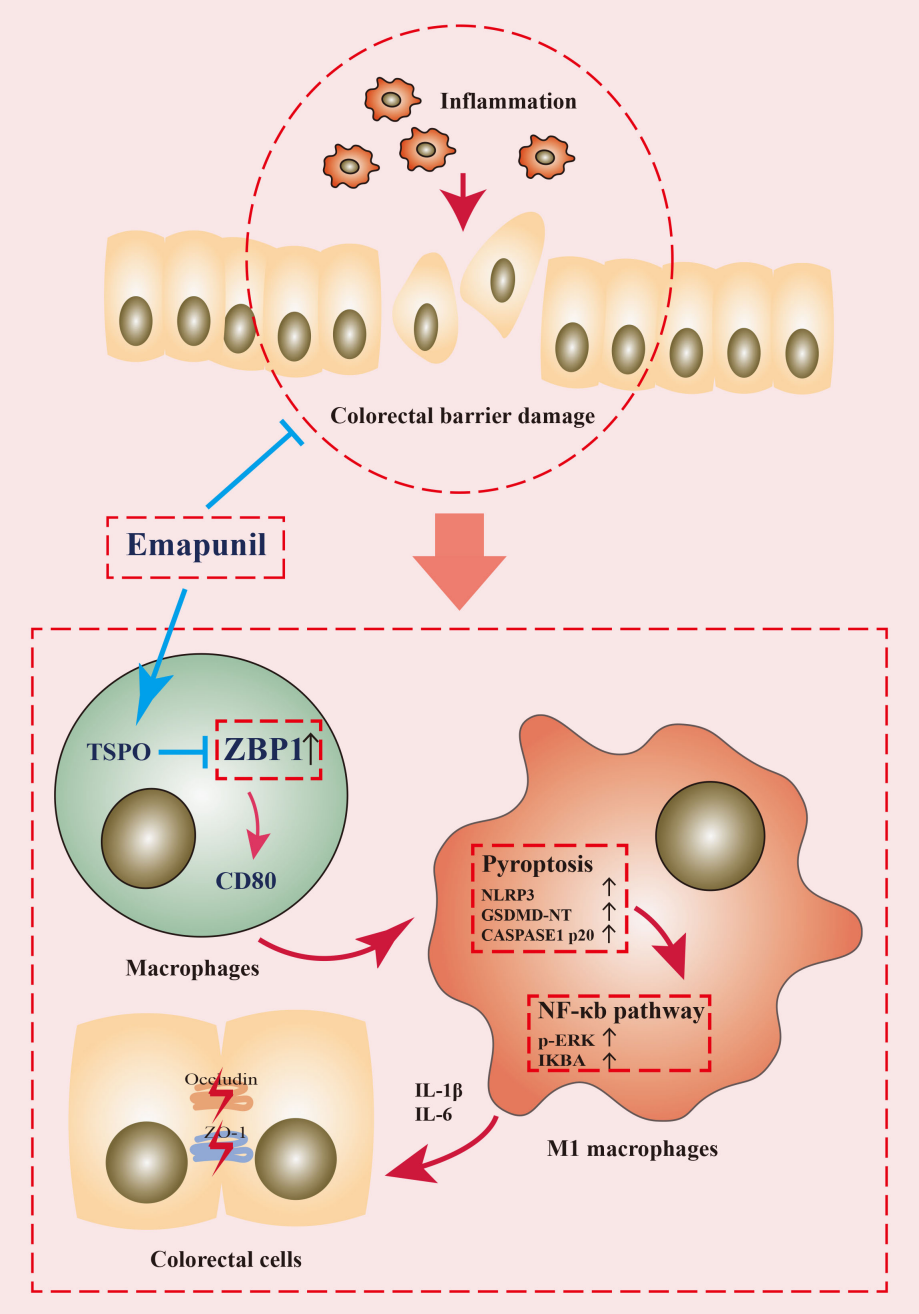
Schematic diagram of the mechanism underlying Emapunil-mediated attenuation of UC. In UC, ZBP1 is abnormally upregulated in colorectal macrophages, which in turn induces pyroptosis and promotes M1 polarization of macrophages while activating the NF-κB signaling pathway. These pathological processes collectively lead to the excessive release of pro-inflammatory cytokines and disruption of intestinal epithelial barrier integrity, ultimately exacerbating colonic inflammation. Emapunil exerts a protective effect against UC by downregulating ZBP1 expression in a TSPO-dependent manner in colorectal macrophages, which subsequently inhibits ZBP1-mediated pyroptosis and M1 polarization, suppresses NF-κB pathway activation, reduces pro-inflammatory cytokine secretion and preserves the integrity of the intestinal epithelial barrier.

In summary, we investigated the crucial role of elevated ZBP1-induced pyroptosis and M1 polarization of macrophages during UC pathogenesis. We found that Emapunil exerted therapeutic effects by inhibiting ZBP1 in a TSPO-dependent manner in the context of UC (**Figure 9**). These findings provide novel mechanistic insights and a potential therapeutic strategy for UC and establish an experimental foundation for the clinical translation of Emapunil in the treatment of UC.

## Data Availability

The raw data supporting the conclusions of this article will be made available by the authors, without undue reservation.

## Supplementary material

Supplementary Figure 1 Validation of THP-1 macrophage differentiation by CD68 mRNA expression upon PMA treatment. Relative mRNA expression of CD68 in THP-1 cells after treatment with 50 ng/mL PMA for 24 hours (n = 6 per group). THP−1 cells were first differentiated into mature macrophages by treatment with 50 ng/mL Phorbol myristate acetate for 24 hours *P < 0.05, **P < 0.01, ***P < 0.001, ns: not significant. Data are presented as mean ± SD. Statistical analyses were conducted using one-way ANOVA followed by Tukey’s *post-hoc* test.

Supplementary Figure 2 Identification of optimal Emapunil concentrations for anti-inflammatory effects in Caco2 and THP-1 cells without cytotoxicity. **(A, B)** CCK−8 assay was performed to assess the cell viability of Caco−2 **(A)** and THP−1 **(B)** cells treated with gradient concentrations of Emapunil (0, 75, 150, 300, 600, 1200, 2400ng/mL) at 0 h, 24 h, and 48 h. **(C)** Relative mRNA expression of CD80, L-1βand IL-6 in THP-1 cells under the indicated concentrations(0, 75, 150, 300, 600ng/mL) of Emapunil. (n = 6 per group). *P < 0.05, **P < 0.01, ***P < 0.001, ns: not significant. Data are presented as mean ± SD. Statistical analyses were conducted using one-way ANOVA followed by Tukey’s *post-hoc* test. **(D)** Relative mRNA expression of CD80, L-1βand IL-6 in THP-1 cells treated with 600ng/mL Emapunil for 0, 6, 12 and 24 h. (n = 6 per group). *P < 0.05, **P < 0.01, ***P < 0.001, ns: not significant. Data are presented as mean ± SD. Statistical analyses were conducted using one-way ANOVA followed by Tukey’s *post-hoc* test.
