# Supplementary figures and images for: Emapunil attenuates ulcerative colitis by suppressing Z-DNA binding protein 1 driven pyroptosis and pro-inflammatory polarization in macrophages
Source: Front Immunol. 2026 May 26;17:1813664. doi: 10.3389/fimmu.2026.1813664 (PMC13246352; doi:10.3389/fimmu.2026.1813664)

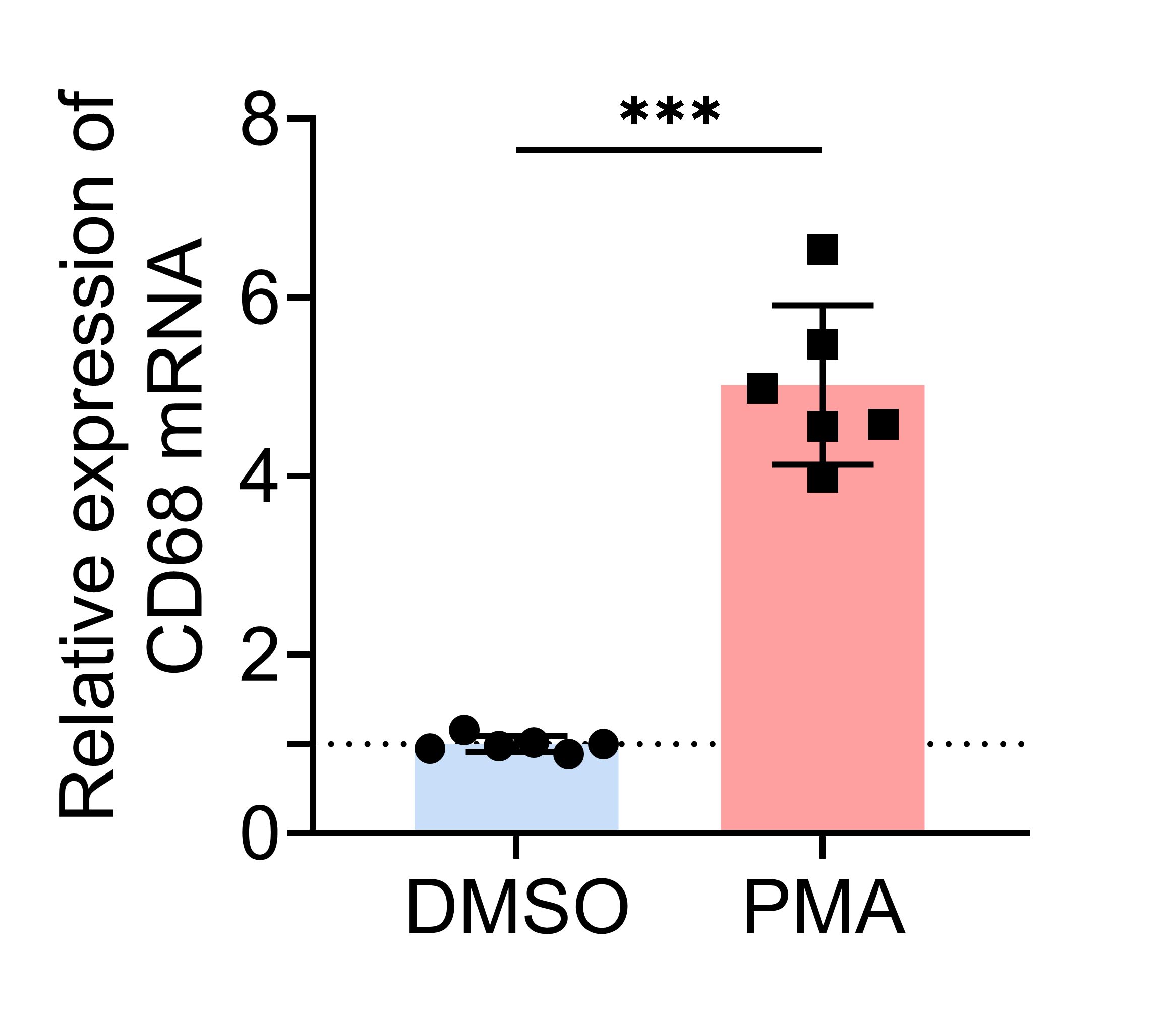

Supplement: Supplementary Figure 1 — Validation of THP-1 macrophage differentiation by CD68 mRNA expression upon PMA treatment. Relative mRNA expression of CD68 in THP-1 cells after treatment with 50 ng/mL PMA for 24 hours (n = 6 per group). THP−1 cells were first differentiated into mature macrophages by treatment with 50 ng/mL Phorbol myristate acetate for 24 hours *P < 0.05, **P < 0.01, ***P < 0.001, ns: not significant. Data are presented as mean ± SD. Statistical analyses were conducted using one-way ANOVA followed by Tukey’s post-hoc test. [file Image1.jpeg]

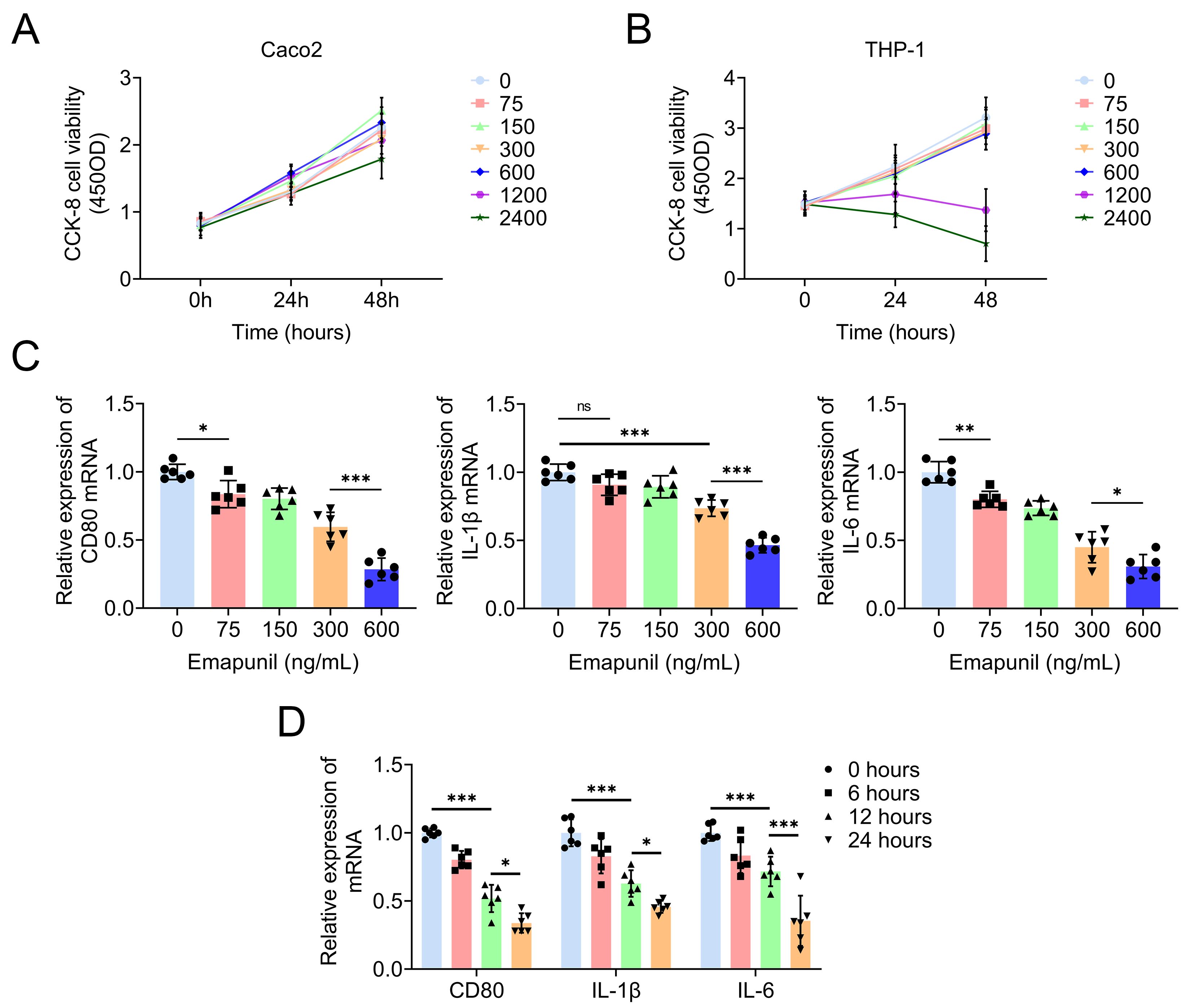

Supplement: Supplementary Figure 2 — Identification of optimal Emapunil concentrations for anti-inflammatory effects in Caco2 and THP-1 cells without cytotoxicity. (A, B) CCK−8 assay was performed to assess the cell viability of Caco−2 (A) and THP−1 (B) cells treated with gradient concentrations of Emapunil (0, 75, 150, 300, 600, 1200, 2400ng/mL) at 0 h, 24 h, and 48 h. (C) Relative mRNA expression of CD80, L-1βand IL-6 in THP-1 cells under the indicated concentrations(0, 75, 150, 300, 600ng/mL) of Emapunil. (n = 6 per group). *P < 0.05, **P < 0.01, ***P < 0.001, ns: not significant. Data are presented as mean ± SD. Statistical analyses were conducted using one-way ANOVA followed by Tukey’s post-hoc test. (D) Relative mRNA expression of CD80, L-1βand IL-6 in THP-1 cells treated with 600ng/mL Emapunil for 0, 6, 12 and 24 h. (n = 6 per group). *P < 0.05, **P < 0.01, ***P < 0.001, ns: not significant. Data are presented as mean ± SD. Statistical analyses were conducted using one-way ANOVA followed by Tukey’s post-hoc test. [file Image2.jpeg]
